# Supplementary material for: A Simple and Scalable Kernel Density Approach for Reliable Uncertainty Quantification in Atomistic Machine Learning
Source: J Phys Chem Lett. 2025 Oct 16;16(42):11081–6. doi: 10.1021/acs.jpclett.5c02595 (PMC12557357; doi:10.1021/acs.jpclett.5c02595)
Supplement: Supplementary file 1 [file jz5c02595_si_001.pdf]

Supporting Information

A Simple and Scalable Kernel Density Approach for Reliable Uncertainty Quantification in Atomistic Machine Learning

Daniel Willimetz[1] and Lukáš Grajciar*[1]

[1]Department of Physical and Macromolecular Chemistry, Charles University, Hlavova 8, Praha 2, 12800, Czech Republic

S1 KDE parameters

The KDE method described in Equation 1 of the main text has two key parameters: (i) the bandwidth of the kernel, h, and (ii) the number of nearest neighbors, k. To illustrate the effect of different choices for these parameters, we use the rMD17 dataset and correlate the error in force prediction for each atom with the KDE score (Figure S1). More details on the rMD17 models and training procedure are provided in Section S3.3.

S1.1 Bandwidth Estimation

The kernel bandwidth h can be estimated using various heuristics. Two commonly used approaches are the Silverman's rule of thumb[1]:

$$h_{\text{Silverman}} = 0.9\,\sigma\,n^{-\frac{1}{5}} \tag{S1}$$

where σ is the standard deviation of all considered nearest-neighbor distances, n is the number of samples.

Silverman's rule can also be formulated using the interquartile range (IQR) instead of the standard deviation, leading to a more robust estimator[1]:

$$h_{\text{Silverman,IQR}} = 0.9\ \min\left(\sigma, \frac{\text{IQR}}{1.34}\right) n^{-\frac{1}{5}} \tag{S2}$$

*Email: lukas.grajciar@natur.cuni.cz

For large datasets, as commonly encountered in training machine learning potentials, the $n^{-\frac{1}{5}}$ scaling often results in bandwidths that are too small to adequately capture the underlying density. Therefore, we additionally investigate other heuristically set bandwidths defined directly from the nearest-neighbor distance distribution:

$$h_{\text{std}} = \sigma, \quad h_{\text{mean}} = \mu, \quad h_{\text{median}} = \tilde{d} \tag{S3}$$

where μ is the mean and $\tilde{d}$ the median of the nearest-neighbor distances.

The performance of these bandwidth selection methods is shown in Figure S1, where the KDE score ρ is correlated with the force error $\hat{F} - F$. The number of nearest neighbors is fixed at $k = 100$.

As expected, the traditional rule of thumb yields bandwidths smaller by approximately one order of magnitude compared to the heuristic alternatives, leading to scores that fail to smoothly cover the full range of uncertainty values. In contrast, the heuristic bandwidths based on σ, μ, or $\tilde{d}$ show comparable performance. For the remainder of this study, we adopt the standard deviation-based bandwidth h_{std}, since it is consistent with the role of σ in the rule of thumb. However, all three heuristic choices lead to similar bandwidth magnitudes and, consequently, similar predictive performance.

This behavior can be further demonstrated by performing the same test for the Pt_5 cluster in the CHA zeolite (Section S3.1). Figure S2 compares the bandwidths obtained using Silverman's rule of thumb (Equation S1) with those from the heuristic standard deviation approach (Equation S3). Using Silverman's rule of thumb, the minimum score, defined as the smallest KDE score across all atomic environments, is close to zero, indicating extrapolation. In contrast, no extrapolation is observed with the NNP ensemble (Figure 2 in the main text), further demonstrating that this bandwidth estimator is unsuitable for the present application.

S1.2 k selection

The parameter k, denoting the number of nearest neighbors, reflects how many neighboring atomic environments from the training database are considered to evaluate the score value for the query environment. To assess the impact of this parameter, we investigated a range of k values from 1 to 2000 (Figure S4). The results show that nearly all tested values of k yield similar correlations, indicating that the precise choice of this parameter is not critical. As a practical rule of thumb, we select $k = 100$, which offers a balance between including multiple relevant environments and maintaining computational efficiency while also smoothly covering the whole range from 0 to 1 (Figure S5. Larger values of k may be chosen depending

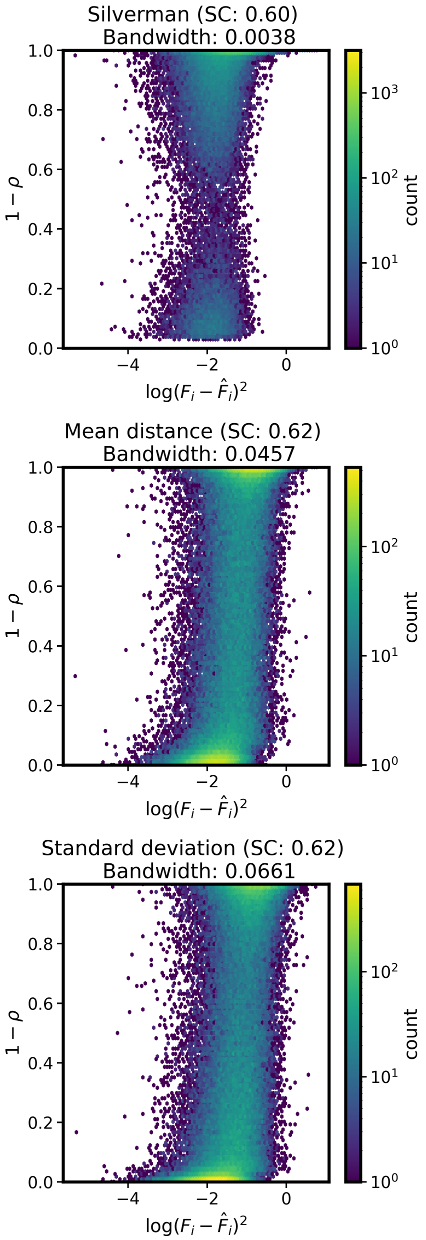

Figure S1: Correlation of the KDE scores ρ and force prediction errors for different kernel bandwidth estimations with the respective Spearman coefficient (SC).[2] The KDE score ρ is correlated with the force error $\hat{F} - F$, where $\hat{F}$ are the reference forces and F are the predicted forces from the trained NNP (Section S3.3). The number of nearest neighbors considered k is fixed at 100.

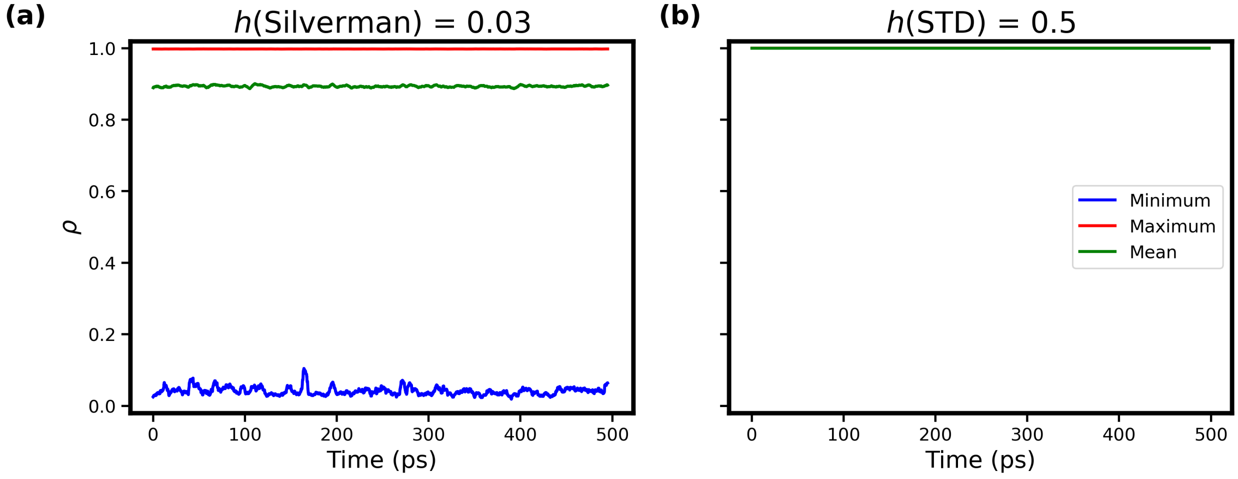

Figure S2: KDE score of the MD simulation of the Pt_5 cluster in CHA zeolite using two bandwidth estimators: (a) Silverman's rule of thumb (Equation S1) and (b) the heuristic standard deviation approach (Equation S3) (mean KDE score overlaps with the minimum and maximum KDE scores).

on the specific application, although they generally (mostly linearly) increase computational cost without substantially improving accuracy (Figure S3)

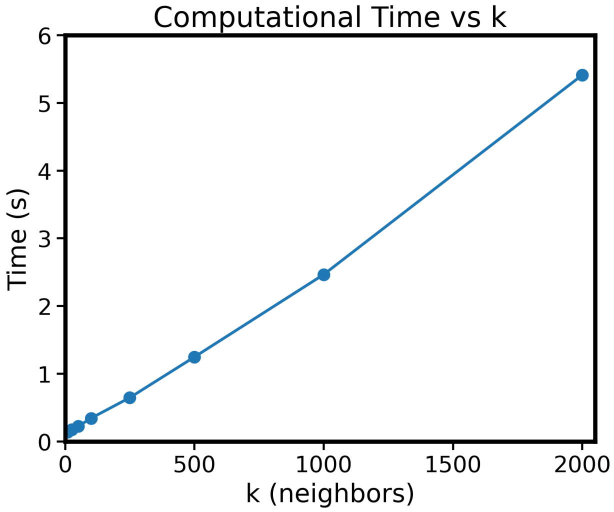

Figure S3: Computational time of KDE score calculations for the rMD17 database as a function of k, showing approximately linear scaling.

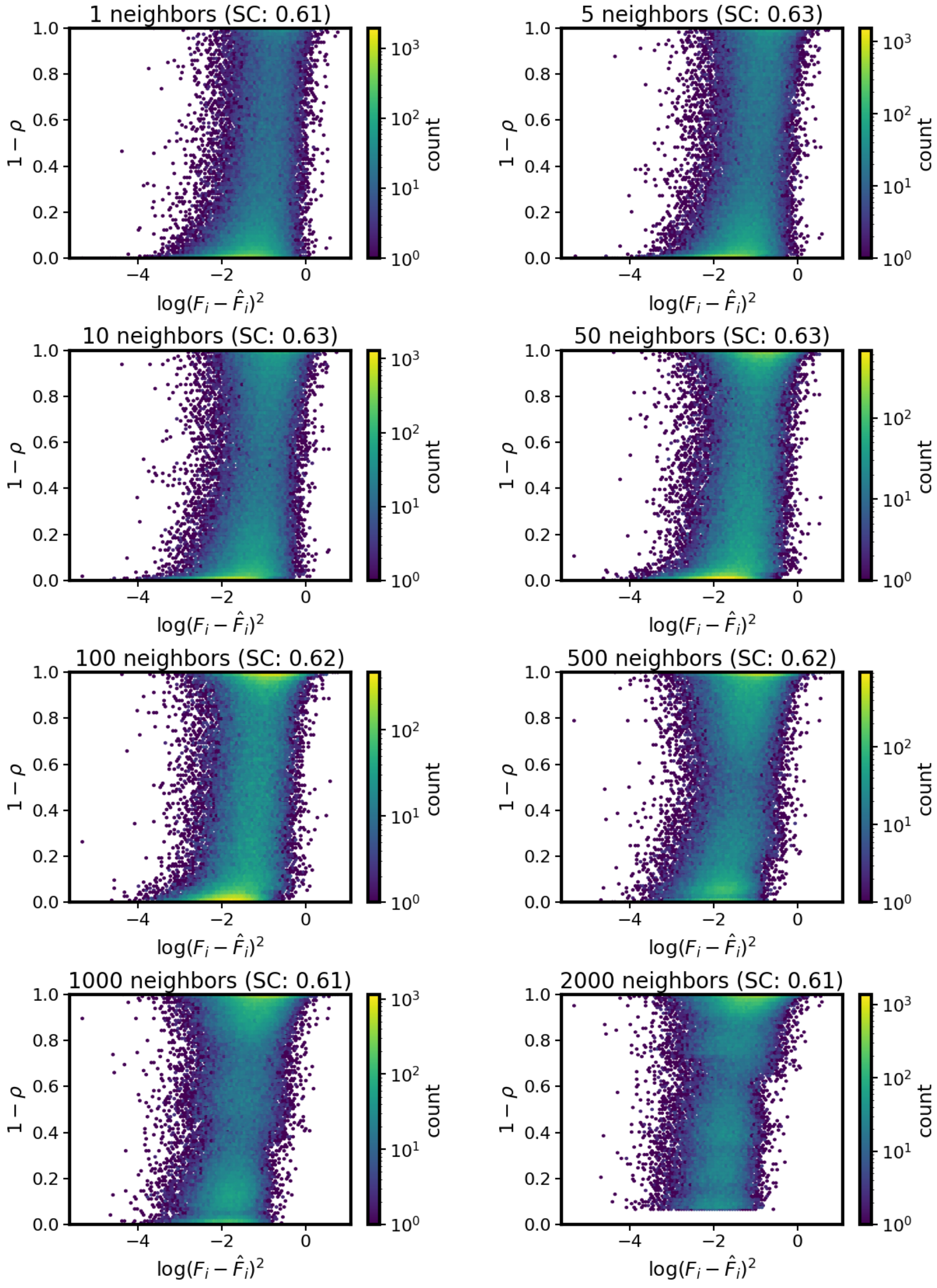

Figure S4: Correlation of the KDE scores ρ and force prediction errors for different the numbers of nearest neighbors k with the respective Spearman coefficients (SC). The kernel bandwidth is fixed at $h = 0.066$

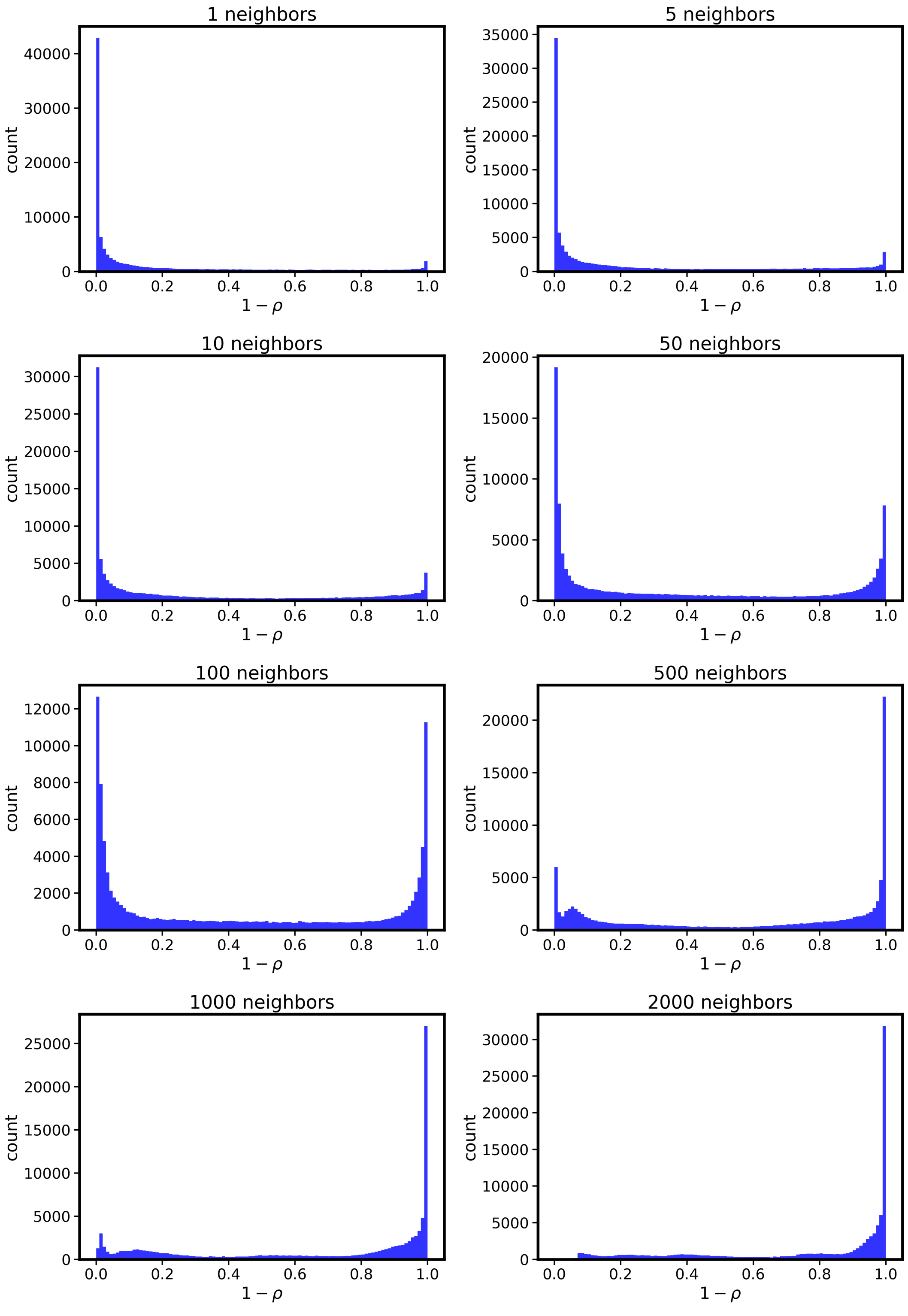

Figure S5: Histograms of KDE scores ρ for different numbers of considered neighbors k (as in Figure S4), illustrating the distribution of ρ values within the range of KDE scores.

S2 Atomic descriptors and PCA

For all case studies involving neural network potentials (1-3), the same atomic descriptors were employed. To construct these descriptors, the small MACE-MP-0a foundational model was used[3], where the invariant descriptors were created using the MACE calculator implemented in Atomic Simulation Environment (ASE).[4] To further improve the efficiency of the method, these 256-dimensional descriptors were subjected to dimensionality reduction via principal component analysis (PCA) using sklearn implementation with the randomized singular value decomposition (SVD) solver.[5] To determine the optimal level of dimensionality reduction, we evaluated the approach using the rMD17 dataset. The results for various reduced dimensions are shown in Figure S6.

From Figure S6, it is evident that correlation deteriorates significantly when the number of dimensions is reduced below 16. Therefore, in all subsequent applications of the MACE atomic descriptors, we fix the dimensionality to 16, thus preserving accuracy while further enhancing computational efficiency.

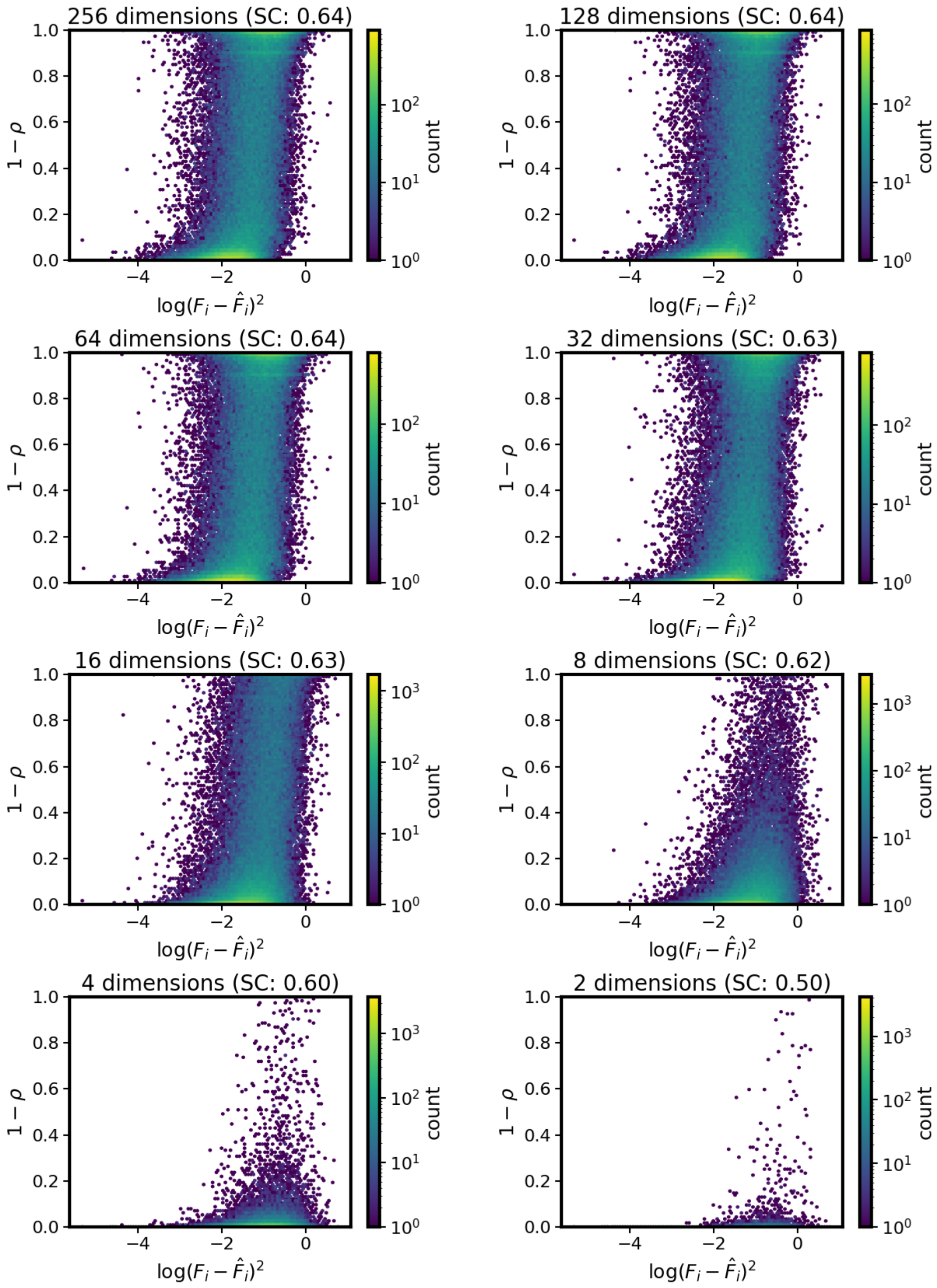

Figure S6: Correlation of the KDE scores ρ and force prediction errors for different degree of dimensionality reduction via PCA for rMD17 dataset with the respective Spearman coefficients (SC) also shown.

S3 Case studies

S3.1 Platinum clusters in silica

The training database was obtained from Benešová et al.[6], which extends the original dataset by Heard et al.[7]. The Pt_5 cluster in CHA was taken from the same study. Although the full molecular dynamics (MD) trajectory spans 25 ns, only the first 500 ps were selected for this case study. The MD simulations were performed at 750 K. In contrast, the Pt_6 cluster on silicatene with a silanol nest was taken from Benešová et al.[6], with the first 500 ps sampled from MD at 2000 K. Representative structures are shown in Figure S7

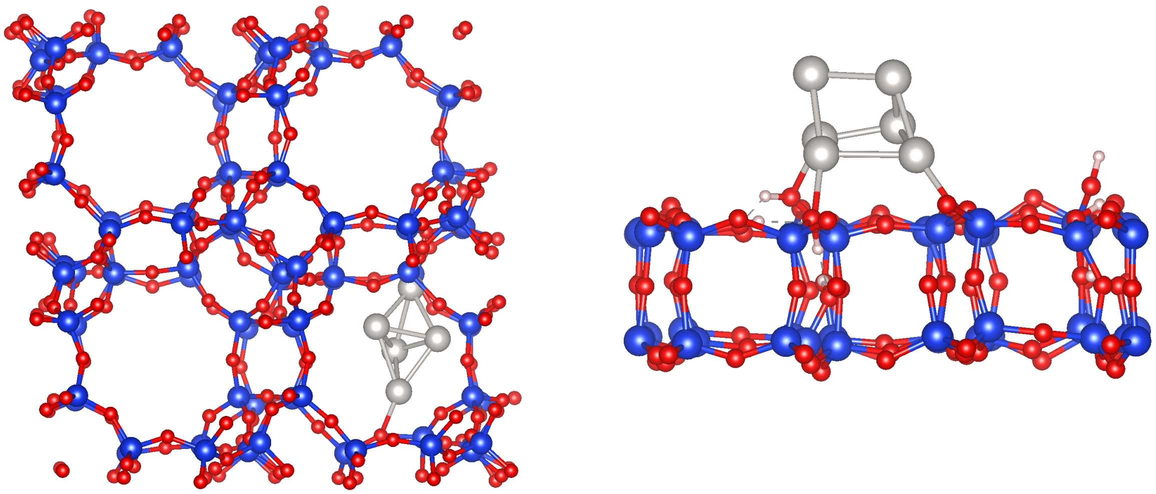

Figure S7: Representative structures for Pt_5 cluster in CHA zeolite (left), and Pt_6 cluster on defective silicatene (right), with Pt atoms in silver, Si in blue, O in red, and H in white.

The KDE parameters were based on the parameters presented in Section S1 using the MP-0a foundational model training database.[3] The resulting KDE bandwidth h is 0.5, which was also used for the H-MFI case study (Section S3.2). Using a universal bandwidth value has an obvious advantage of being transferable and interpretable, with a minimum KDE score below 0.5 signalling a potential extrapolation. The minimum score is defined as the smallest score out of all atomic environments in a given test structure.

To demonstrate both that our approach for bandwidth selection is reasonable and that the MACE-MP0 descriptors are capable of producing results comparable to model-specific descriptors, we evaluated the performance of the method using also the SchNet descriptors in Figure S8. The SchNet descriptors were reduced from their original 128 dimensions to 16 dimensions using the same PCA approach. The bandwidth for the SchNet descriptors was determined following the same procedure described above for the MACE-MP0 training

database, with the structures filtered to include only the relevant elements (Si, Al, O, H, Pt). The resulting bandwidth for the PCA-reduced SchNet descriptors was found to be $h = 98$.

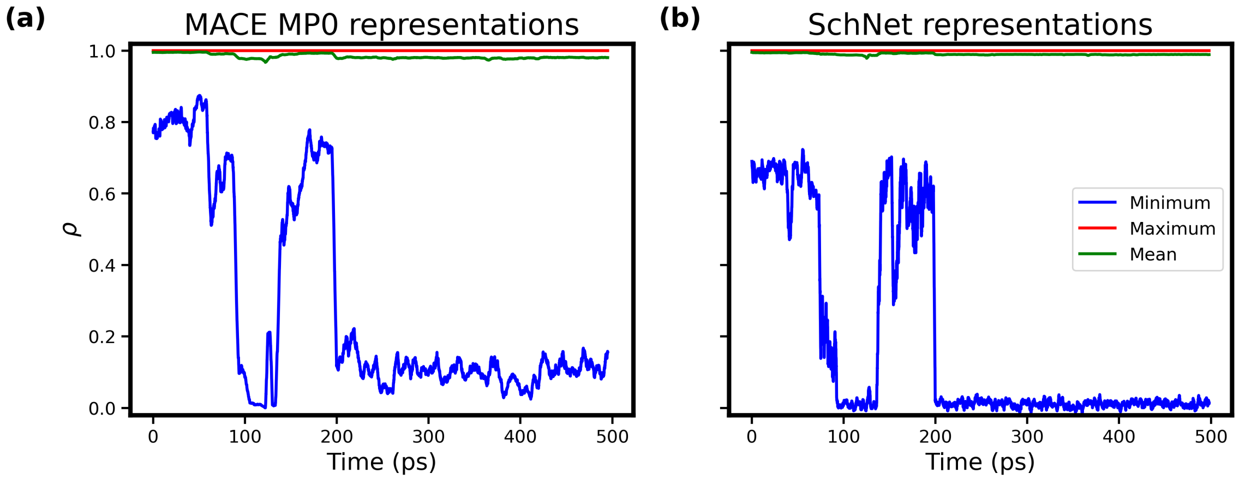

Figure S8: Comparison of the KDE-based score evaluated for the Pt_6 cluster on silicatene using different atomic descriptors: (a) generic MACE MP0 descriptors, and (b) model-specific SchNet descriptors.

Figure S8 shows that the generic MACE-MP0 descriptors reproduce the trends observed with the SchNet descriptors for the MD simulation of Pt_6 cluster on silicatene. This confirms that MACE descriptors can effectively replace model-specific descriptors, at least in this case study.

S3.2 MP0 foundational model for H-MFI

Initial structures of MFI were generated to sample different Si/Al ratios and water loadings. Two models were considered: (i) Si/Al = 95 and (ii) Si/Al = 11. The initial structures in the MD simulation are shown in Figure S9. In both cases, all aluminum atoms were placed at the T5 sites, and the proton was placed on a neighboring oxygen to form a Brønsted acid site. One water molecule per aluminum atom was added to represent different total water loadings. Molecular dynamics simulations were performed using neural network potentials trained on a comprehensive zeolite database,[8] ensuring realistic dynamics. The simulations were carried out at 350 K using a Nosé-Hoover thermostat[9] for a duration of 100 ps with a time step of 0.5 fs. The MD simulation code was taken from the ASE python package.[4] The KDE score was calculated using exactly the same approach as in the case study described in the previous Section S3.1.

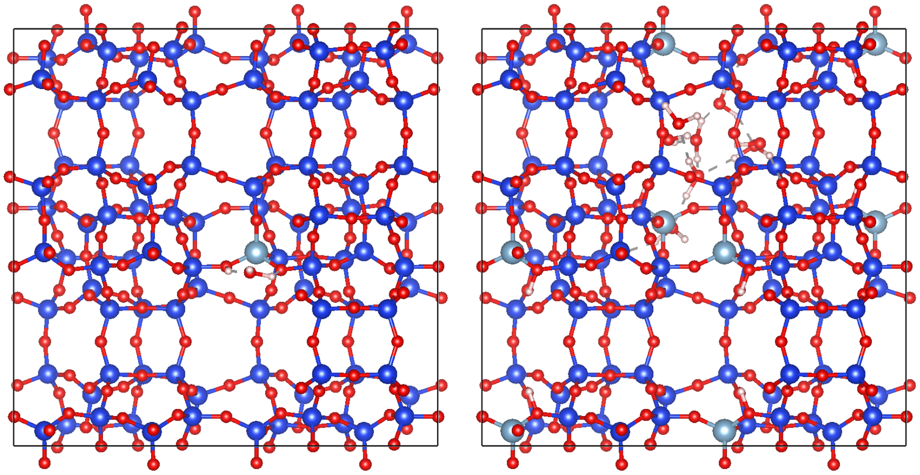

Figure S9: Initial structures used for the MD simulations of H-MFI zeolite. Left: Si/Al = 95 (1 Al per unit cell) with 1 water molecule per unit cell. Right: Si/Al = 11 (8 Al per unit cell) with 8 water molecules per unit cell.

S3.3 rMD17

The entire rMD17 database was divided into training and testing sets using the train/test split provided in the published database.[10] For the query-by-committee approach, five models were created using the same architecture hyperparameters as used for the MACE-MP-0a-small model. The cutoff was set to 6 Å, with 128 channels, and only invariant features were used. For the KDE method, the bandwidth was set to 0.066 and k to 100, as described in Section S1. The descriptors used to compute the KDE score were generated by the MACE-MP-0a-small foundational model and subsequently reduced to 16 dimensions using principal component analysis (PCA).

Additionally, the descriptors from the MACE model trained from scratch can also be used to calculate the KDE score, yielding similar results to the descriptors from the MACE-MP0 foundational model (Figure S10). The bandwidth obtained by the standard deviation of nearest neighbor distances also remains similar with $h = 0.058$.

S3.4 ^{27}Al NMR in zeolites

The zeolite structures were prepared by substituting one silicon atom with an aluminum atom at the T1 site, with five water molecules placed near the aluminium. Molecular dynamics simulations were performed at 350 K for 1 ns with a time step of 0.5 fs using previously trained neural network potentials[8]. The MD was carried out in the NVT ensemble using the ASE python package[4] and the Nosé-Hoover thermostat.[9] Subsequently, 50 snapshots

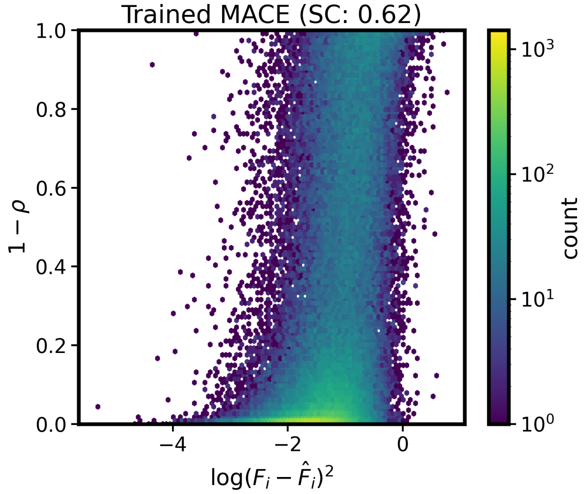

Figure S10: Correlation of the KDE scores ρ and force prediction errors using descriptors from the MACE model trained from scratch. The Spearman coefficient for this correlation (SC) is 0.62.

from the MD simulation were sampled for DFT NMR calculations. The NMR calculations were carried out with CASTEP 24.1[11] at the gamma point, using the PBESOL exchange-correlation functional[12] and a plane-wave cutoff of 700 eV. This same setup was used in our previous publications.[13;14] The SOAP descriptors were generated by the python package DScribe 1.2.2[15] with the following parameters:

```
species = 1 8 11 13 14
r_cut = 5.
n_max = 6
l_max = 6
sigma = 0.5
average = "off"
```

To evaluate how far the dimensionality of the high-dimensional SOAP descriptors can be reduced while still retaining a informative KDE score, we applied PCA to compress the descriptors from their original size (5500 dimensions) down to as few as a couple of dimensions. The tests (Figure S11) show that for SOAP descriptors, a reduction to around 16 dimensions is sufficient to preserve the relevant information, consistent with what we observed earlier for MACE descriptors.

The bandwidth for the KDE calculation was determined following the same procedure described above. The standard deviation of the nearest neighbor distances yielded $h \approx$ 50,000. Table S1 compares the average scores obtained using the original and PCA-reduced

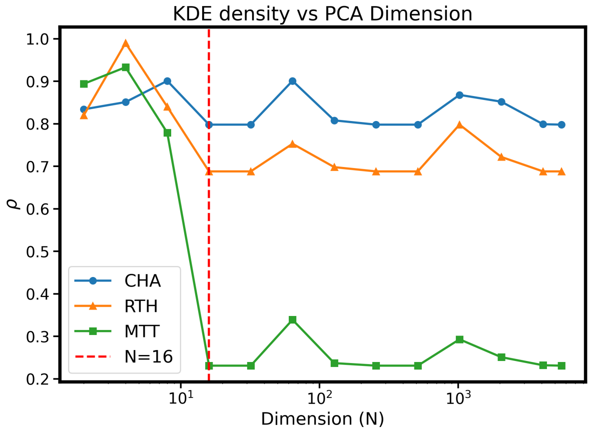

Figure S11: KDE scores for the three tested zeolites (CHA, MTT, RTH) obtained from Al-centered SOAP descriptors after PCA dimensionality reduction.

descriptors. The bandwidth remains roughly the same for both cases (49,189 for the original descriptor and 48,709 for the reduced descriptor), and the average score is also approximately unchanged. This shows that the SOAP descriptors contain significant redundancy.

Table S1: Comparison of average scores for PCA-reduced and original SOAP descriptors with their dimensions.

Zeolite	MAE (ppm)	PCA-SOAP (16)	SOAP (5470)
CHA	0.60	0.81	0.80
MTT	1.86	0.24	0.23
RTH	0.72	0.68	0.69

S4 Computational time

To test computational costs, we select the Pt_6 cluster on silicatene zeolite and the training database from Benešová et al.[6], as these represent the largest test structure and training database considered in this study. The atomic descriptors were generated using the MACE-MP0a foundational model, which by default has 256 dimensions. The evaluation procedure for atomic environments was divided into three main computational components: descriptor generation, PCA dimensionality reduction, and KDE-based score estimation. All calculations were performed using a single Tesla T4 GPU and one CPU core.

- **Descriptor Generation:** Descriptors were obtained via the forward pass of the MACE foundational model for each atomic environment. The computational cost

was measured at 197 ms per 1000 atoms, making this the most time-consuming step. This time is relevant to descriptor generation for both atoms of the reference dataset and the test (i.e., query) atoms. For the training database of 4 million atomic environments, the descriptor generation takes approximately 13 minutes. However, the generation of descriptors for the reference dataset is done only once upfront, with the descriptors saved and loaded for the KDE score calculation. Hence, for the KDE score calculation, only descriptors of the query atoms are generated on the fly.

- **PCA Transformation:** Dimensionality reduction was applied to the descriptors to accelerate subsequent calculations and to reduce the size of loaded descriptors (from 13 GB to 800 MB for 4 million atoms) to save both GPU and RAM memory. This step was highly efficient, requiring only 0.4 ms per 1000 atoms, and its contribution to the overall computational time was negligible.
- **KDE score Calculation:** The KDE scores were computed via a Gaussian kernel using FAISS[16]. This step required 54 ms per 1000 atoms, which is significant but still substantially faster than descriptor generation. The KDE score calculation increases linearly with the size of the training set (Figure 1).

The PCA transformation effectively reduces descriptor dimensionality at minimal cost, while descriptor generation, if obtained using a message-passing neural network like MACE, is expected to remain the primary computational bottleneck in large-scale evaluations.

S5 Relation to the spilling factor

The spilling factor introduced by Miwa and Ohno[17] is defined as

$$s_k = 1 - \sum_{k_B=1}^{N_B} \sum_{k'_B=1}^{N_B} K(\mathbf{X}_k, \mathbf{X}_{k_B})\, K^{-1}(\mathbf{X}_{k'_B}, \mathbf{X}_{k_B})\, K(\mathbf{X}_{k'_B}, \mathbf{X}_k), \tag{S4}$$

where $K(\mathbf{X}_i, \mathbf{X}_j)$ denotes the normalized similarity kernel between structures $\mathbf{X}_i$ and $\mathbf{X}_j$, and the summations run over the N_B training data points. If one assumes that the basis functions are orthogonal, i.e.

$$K^{-1}(\mathbf{X}_{k'_B}, \mathbf{X}_{k_B}) = \delta_{k'_B,k_B}, \tag{S5}$$

the expression simplifies to

$$1 - s_k = \sum_{k_B=1}^{N_B} |K(\mathbf{X}_k, \mathbf{X}_{k_B})|^2 . \tag{S6}$$

By further adopting a Gaussian kernel of the form

$$K(\mathbf{X}_k, \mathbf{X}_{k_B}) = \mathcal{N} \exp\left(-\frac{\|\mathbf{X}_k - \mathbf{X}_{k_B}\|^2}{2\tilde{h}^2}\right), \tag{S7}$$

with normalization constant $\mathcal{N}$ and $\tilde{h}^2 = h^2/2$, one obtains an expression closely related to the KDE score (Eq. (1) in the main text). This demonstrates that the KDE score can be regarded as a simplified form of the spilling factor under the orthogonality assumption.

To assess the validity of the orthogonality assumption (Eq. S5), we examined the kernel matrix for PCA-reduced (16-dimensional) MACE-MP0 descriptors from the rMD17 dataset. The off-diagonal elements were found to be several orders of magnitude smaller than the diagonal ones (typically below 10^{-4}, with only very rare cases reaching 10^{-2}), confirming that the kernel matrix is nearly diagonal. This validates the approximation made in Eq. S5, even for chemically similar environments.

Next, we directly compared the spilling scores and the KDE score for 100 randomly selected structures from the rMD17 dataset. As shown in Figure S12, the two measures correlate very well, with both Pearson and Spearman coefficients exceeding 0.95.

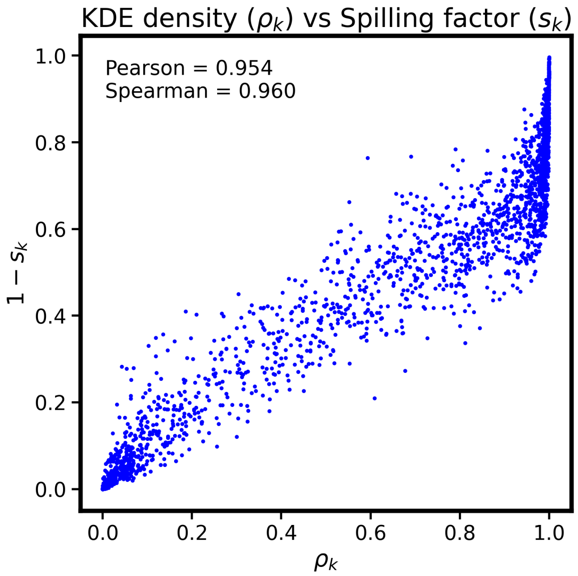

Figure S12: Comparison between the KDE score (ρ_k) and the spilling score (s_k) for 100 randomly selected structures of the rMD17 dataset. Spilling score was normalized so that the maximum value is 1. Both Pearson and Spearman correlation coefficients exceed 0.95.

While the spilling factor and KDE score yield nearly identical results, the computational costs differ dramatically. The spilling factor requires pairwise kernel evaluations between all training points, resulting in quadratic scaling with database size and substantial memory demand. Even when restricted to the 100 closest training points, the spilling factor requires on-the-fly distance matrix construction for every query, making it impractical for large databases.

In contrast, the KDE score scales linearly with the number of training points and can be evaluated efficiently using GPU-accelerated nearest-neighbor search. Given the strong correlation between the two measures, KDE score thus provides a validated and computationally tractable alternative to the spilling factor.

References

[1] Silverman, B. W. *Density Estimation for Statistics and Data Analysis*; CRC Press, 1986.

[2] Dodge, Y. *The Concise Encyclopedia of Statistics*; Springer New York, 2008; pp 502–505.

[3] Batatia, I. et al. A foundation model for atomistic materials chemistry. *arXiv* **2024**, DOI: arxiv:2401.00096.

[4] Larsen, A. H. et al. The atomic simulation environment—a Python library for working with atoms. *Journal of Physics: Condensed Matter* **2017**, *29*, 273002.

[5] Pedregosa, F. et al. Scikit-learn: Machine Learning in Python. *Journal of Machine Learning Research* **2011**, *12*, 2825–2830.

[6] Benešová, T.; Pokorná, K.; Erlebach, A.; Heard, C. Mobility and Sintering of Silica-Supported Platinum Clusters via Reactive Neural Network Potentials. *ChemRxiv* **2025**, DOI: 10.26434/chemrxiv–2025–tjz1c.

[7] Heard, C. J.; Grajciar, L.; Erlebach, A. Migration of zeolite-encapsulated subnanometre platinum clusters *via* reactive neural network potentials. *Nanoscale* **2024**, *16*, 8108–8118.

[8] Erlebach, A.; Šípka, M.; Saha, I.; Nachtigall, P.; Heard, C. J.; Grajciar, L. A reactive neural network framework for water-loaded acidic zeolites. *Nature Communications* **2024**, *15*, 4215.

[9] Evans, D. J.; Holian, B. L. The Nose–Hoover thermostat. *The Journal of Chemical Physics* **1985**, *83*, 4069–4074.

[10] Christensen, A. S.; von Lilienfeld, O. A. On the role of gradients for machine learning of molecular energies and forces. *arXiv* **2020**, DOI: arxiv:2007.09593.

[11] Joyce, S. A.; Yates, J. R.; Pickard, C. J.; Mauri, F. A first principles theory of nuclear magnetic resonance J-coupling in solid-state systems. *The Journal of Chemical Physics* **2007**, *127*, 204107.

[12] Perdew, J. P.; Ruzsinszky, A.; Csonka, G. I.; Vydrov, O. A.; Scuseria, G. E.; Constantin, L. A.; Zhou, X.; Burke, K. Restoring the Density-Gradient Expansion for Exchange in Solids and Surfaces. *Physical Review Letters* **2008**, *100*, 136406.

[13] Willimetz, D.; Martınez-Ortigosa, J.; Brako-Amoafo, D.; Grajciar, L.; Vidal-Moya, A.; Bornes, C.; Sarou-Kanian, V.; Erlebach, A.; Rey, F.; Blasco, T.; Heard, C. Aluminum Siting in Zeolite RTH From a Combined Machine Learning - NMR Approach. *ChemRxiv* **2025**, DOI: 10.26434/chemrxiv–2025–1p3dj.

[14] Willimetz, D.; Erlebach, A.; Heard, C. J.; Grajciar, L. 27Al NMR chemical shifts in zeolite MFI via machine learning acceleration of structure sampling and shift prediction. *Digital Discovery* **2025**, *4*, 275–288.

[15] Himanen, L.; Jäger, M. O. J.; Morooka, E. V.; Federici Canova, F.; Ranawat, Y. S.; Gao, D. Z.; Rinke, P.; Foster, A. S. DScribe: Library of descriptors for machine learning in materials science. *Computer Physics Communications* **2020**, *247*, 106949.

[16] Johnson, J.; Douze, M.; Jégou, H. Billion-scale similarity search with GPUs. *IEEE Transactions on Big Data* **2019**, *7*, 535–547.

[17] Miwa, K.; Ohno, H. Molecular dynamics study on β-phase vanadium monohydride with machine learning potential. *Physical Review B* **2016**, *94*.
