# Supplementary material for: A Simple and Scalable Kernel Density Approach for Reliable Uncertainty Quantification in Atomistic Machine Learning
Source: J Phys Chem Lett. 2025 Oct 16;16(42):11081–6. doi: 10.1021/acs.jpclett.5c02595 (PMC12557357; doi:10.1021/acs.jpclett.5c02595)
Supplement: Supplementary file 2 [file jz5c02595_si_002.pdf]

jz-2025-02595c.R1

Name: Peer Review Information for "A Simple and Scalable Kernel Density Approach for Reliable Uncertainty Quantification in Atomistic Machine Learning"

First Round of Reviewer Comments

Reviewer: 1

Comments to the Author

Journal: The Journal of Physical Chemistry Letters Manuscript ID : jz-2025-02595c Original

Submission Date: 21-Aug-2025 Title : "A Simple and Scalable Kernel Density Approach for

Reliable Uncertainty Quantification in Atomistic Machine Learning"
Author(s): Willimetz, Daniel; Grajciar, Lukáš

Extrapolation beyond the training data is a serious problem commonly occurring in machine learning (ML)-based simulations and almost always leads to a reduction in prediction quality. Unless unphysical configurations are produced, the problem is often difficult to detect, especially if the simulation is performed by a researcher without in-depth knowledge of machine learning. In the submitted work, the authors address this problem by proposing a computationally inexpensive measure that enables the detection of extrapolative regime during ML-based simulations. Given the rapidly growing interest in the use of ML techniques in theoretical chemistry, I am convinced that the submitted work will be of great interest to readers of The Journal of Physical Chemistry Letters. Before I can recommend the manuscript for publications, the following points should be addressed:

1.) The proposed measure ρ_k is closely related to the spilling factor:

$$s_k = 1 - \sum^{N_B} \sum^{N_B} K(\boldsymbol{X}_k, \boldsymbol{X}_{k_B}) K^{-1}(\boldsymbol{X}_{k'_B}, \boldsymbol{X}_{k_B}) K(\boldsymbol{X}_{k'_B}, \boldsymbol{X}_k)$$

$k_B{=}1\ k'_B{=}1$

introduced by Miwa and Ohno DOI: 10.1103/PhysRevB.94.184109 (see also Jinnouchi et al. DOI: 10.1103/PhysRevB.100.014105) to serve a similar purpose as ρ_k. In equation above, $K(\boldsymbol{X}_i, \boldsymbol{X}_j)$ is a normalized similarity kernel between structures $\boldsymbol{X}_i$ and $\boldsymbol{X}_j$, and summations are over training set basis functions. Under assumption that all $\boldsymbol{X}_{k'_B}, \boldsymbol{X}_{k_B}$ pairs are orthogonal

(i.e., $K^{-1}(\boldsymbol{X}_{k'_B}, \boldsymbol{X}_{k_B}) = \delta_{k'_B,k_B}$), we obtain

$$1 - s_k = \sum_{k_B=1}^{N_B} \sum_{k'_B=1}^{N_B} K(\boldsymbol{X}_k, \boldsymbol{X}_{k_B})\delta_{k'_B,k_B} K(\boldsymbol{X}_{k'_B}, \boldsymbol{X}_k) = \sum_{k_B=1}^{N_B} |K(\boldsymbol{X}_k, \boldsymbol{X}_{k_B})|^2$$

Setting

$$K(\boldsymbol{X}_k, \boldsymbol{X}_{k_B}) = \mathcal{N} e^{-(\boldsymbol{X}_k - \boldsymbol{X}_{k_B})^2/2\tilde{h}^2}$$

with $\mathcal{N}$ being normalization constant and $\tilde{h}^2 = h^2/2$, we obtain eq. 1 in the manuscript. Since, based on this analysis, ρ_k can be viewed as a simplified version of $1 - s_k$, the authors should (i) acknowledge the relation between ρ_k and s_k, (ii) explain why the simplifications (especially ignoring redundancy/non-orthogonality of training data) are reasonable - ideally support their arguments with numerical results where performance of ρ_k and $1 - s_k$ would be compared, and (iii) discuss the relative benefits of ρ_k over $1 - s_k$.

2.) Is the normalization in eq. 1 correct? Assume a 1D case with $\mathcal{N}_k$ =100 training points that are separated by a distance much larger than h and assume we set $q = x_1$. In this case, the only non-vanishing exponential term (that for $i = 1$) equals 1 and thus
ρ_k =0.01.

3.) The phrase “h is the kernel band-width, taken as the standard deviation of distances within the training set” on P. 2, l.53-55 is redundant because the next sentence “bandwidth, evaluated for each reference database separately, was calculated as the standard deviation of k nearest-neighbor distances averaged over all reference database entries,” contains the same information.

4.) The correlation between $\log(1 - \varrho)$ and $\log(F_i - \tilde{F}_i)^2$ demonstrated in Fig. 4 and multiple figures in SI is in fact not very convincing. From what I see, there are many structures with ϱ close to 1 and large $(F_i - \tilde{F}_i)^2$ or, in contrast, with ϱ close to 0 and

small $(F_i - \tilde{F}_i)^2$. The authors should explain why they consider such a correlation (with correlation coefficients around 0.6) to be satisfactory. It would be useful if the authors could show that the reduced correlation is not due to the simplifications mentioned in my point 1. To this end, the authors could simply perform a similar correlation analysis with the spilling factors with Gaussian kernel and show that the correlation coefficient does not improve significantly.

5.) Fig. 4, left panel – what is ϱ (without index) here? – average of ρ_k, minimal value of ρ_k, or simply ρ_k(i.e., one point in graph represents one atom)?

6.) In table 1 the authors show how their KDE density changes over a small series of similar systems described by a ML models of different quality. In this case, the KDE density values vary between 0.25 (bad description) to 0.85 (good description). Is it possible to define a threshold for a reasonable description? I know that this is not easy to say but a simple rule of thumb like 'any value below 0.5 is a clear indication of poor quality of simulation' would be useful for practical applications.

7.) SI, Figure S1, S4, S5 and S9 – I do not think it is "Performance" what these plots really show (maybe correlation between ϱ[illegible]and error in predicted forces?). Also, meaning of "SC" (Spearman coefficient?) is not explained.

8.) Figure S2, left panel - "maximum" and "minimum" curves are not shown.

Reviewer: 2

Comments to the Author

Overview: This work provides a model-agnostic method for identifying out-of-distribution atomic environments based on representation in the training data. The work provides a similarity value based on the local density ρ, where a value of 1 indicates good representation in the training set and 0 indicates poor representation in the training set. The authors state in the supporting information that a value below 0.5 typically indicates that the model is extrapolating rather than interpolating. Notably, this method is performed on atomic environments to give a ρ for each atom. The method is sound, the text is well written, and the conclusions are supported by the results.

Comment 1: I wouldn't call ρ an uncertainty, as uncertainties tend to put bounds around the output value (such as energy or forces). ρ is certainly a metric useful for identifying out-of-distribution atomic environments. In my opinion, ρ should be identified as such, rather than as a metric for uncertainty. Implementing this change would require a good amount of editing of the text and a change of title, but would not affect any of the results or conclusions of the work.

Comment 2: The supporting information provides information on formulating the kernel bandwidth using the interquartile range (IQR) instead of the standard deviation, along with a brief discussion on why the standard deviation is preferred in this work. It would be useful to include a sentence or two in the main text pointing to the discussion in the SI to justify the use of standard deviation over IQR.

Comment 3: The value of k-nearest neighbors and number of dimensions for PCA were explored for reducing the MACE-MP0 descriptors of the rMD17 dataset. When moving to new descriptors and/or datasets, would the value of k and number of dimensions need to be tuned? It would be helpful to show a similar examination, at least for number of dimensions for the reduction of Al-centered SOAP descriptors since the original number of dimensions is so much higher than that of the MACE-MP0 descriptor. Though a comparison of PCA-SOAP (16) and SOAP (5470) is given in Table S1, a plot of descriptor size vs average score (once more values were explored) would better indicate the effect of reducing the number of dimensions. It would also be interesting to see if the effect is the same for all 3 zeolites.

Comment 4: How is the optimal k determined? From Fig. S4, it appears that there are two dense regions of ρ near 0 and 1 at k=100. Is this the main criterion? If so, a histogram of ρ would be more useful to display than comparison against force errors.

Author's Response to Peer Review Comments:

General response to Reviewers:

We thank the Reviewers for their suggestions and constructive criticism. We have revised the manuscript (and Supplementary Information) following the Reviewers' suggestions.
Below, we have responded to each point (in black) made by the reviewers with our comments (blue). In addition, we have provided the manuscript file with the changes explicitly highlighted in yellow.

Reviewers' comments:

Reviewer: 1

1) The proposed measure ρ_k is closely related to the spilling factor ...

Since, based ρ_k can be viewed as a simplified version of 1- s_k, the authors should (i) acknowledge the relation between ρ_k and s_k, (ii) explain why the simplifications (especially ignoring redundancy/non-orthogonality of training data) are reasonable - ideally support their arguments with numerical results where performance of ρ_k and 1-s_k would be compared, and (iii) discuss the relative benefits of ρ_k over 1-s_k.

We thank the reviewer for a very relevant point raised and for providing the relevant references. We have now acknowledged this connection briefly in the manuscript and added a full section (Section S5) related to comparison between the KDE density and spilling factor in of the Supporting Information, following the points proposed by the reviewer. Below we shortly summarize the results of our analysis.

To assess the validity of the orthogonality assumption, we calculated the kernel matrix of training set descriptors for the rMD17 dataset. The off-diagonal elements were found to be negligible, as the 99.9th percentile is below 1% of the corresponding diagonal elements. This indicated that the kernel matrix is nearly diagonal (even in this rather densely covered reference dataset) and supports the orthogonality assumption made.

We further compared the spilling factor and KDE density for 100 randomly selected structures from the rMD17 dataset. As shown in Figure below (and also included as a Figure S12 in SI), the two measures correlate very strongly, with Pearson and Spearman coefficients exceeding 0.95. This demonstrates that the KDE density reproduces the spilling factor. Lastly, the evaluation of KDE density is more computationally efficient scaling linearly with the database size (Figure 1b), in contrast to spilling factor scaling quadratically (both in memory and CPU time), limiting its applicability to large datasets.

More details are presented in Section S5 of the Supporting Information.

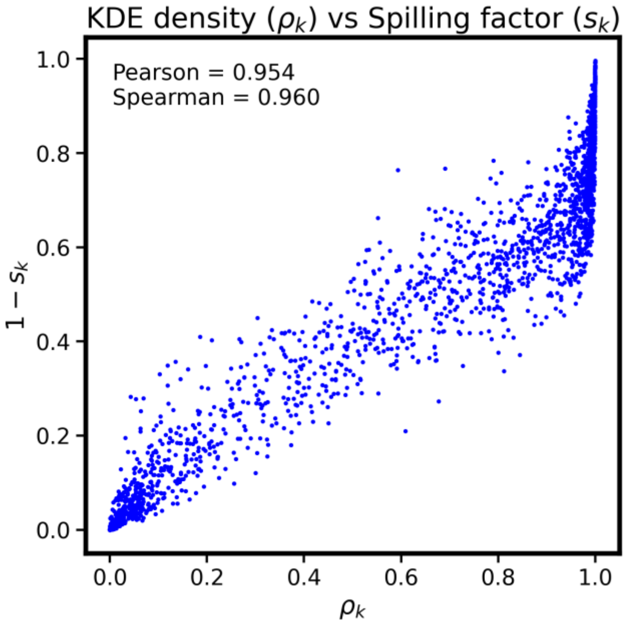

2) Is the normalization in eq. 1 correct? Assume a 1D case with $\mathcal{N}_k$= 100 training points that are separated by a distance much larger than h and assume we set q=x_1. In this case, the only non-vanishing exponential term (that for i=1) equals 1 and thus ρ_k=0.01.

That is correct, and this was done intentionally. If the query is represented in the database by only a single training point while the other points are far away, this is not sufficient for a reliable machine learning prediction (10.1016/j.aca.2012.11.007). Furthermore, upon further review, we have decided to rename the KDE density to KDE score, as the normalization does not include standard h^(d), where d is the dimensionality. For high dimensional application, this term would be impractical and we use the normalization presented in MS as it is also more efficient than calculating true density. This sentence was added to the MS for clarification: "Although this expression does not yield a globally normalized KDE density, it provides a consistent local similarity measure. In standard KDE, the density includes a factor $\frac{1}{h^d}$, where d is the descriptor dimensionality. For high-dimensional data this term becomes impractical, so here the normalization is approximated by using a Gaussian kernel with maximum value 1 and scaling by $\frac{1}{k}$, resulting in the local KDE score limited to values between 0 and 1 by construction. This scaling also reflects the expectation that multiple similar environments are required for a reliable prediction, with a single nearest neighbor ($k=1$) being generally insufficient to produce a meaningful score..[18]"

3) The phrase “h is the kernel band-width, taken as the standard deviation of distances within the training set” _on P. 2, l.53-55 is redundant because the next sentence “bandwidth, evaluated for each reference database separately, was calculated as the standard deviation of k nearest-neighbor distances averaged over all reference database entries,” contains the same information.

We thank the reviewer for noticing the redundancy and we have modified the text to remove it.

4) The correlation between $\log(1-\varrho)$ and $\log(F_i - \hat{F}_i)^2$ demonstrated in Fig. 4 and multiple figures in SI is in fact not very convincing. From what I see, there are many structures with ϱ close to 1 and large $(F_i - \hat{F}_i)^2$ or, in contrast, with ϱ close to 0 and small $(F_i - \hat{F}_i)^2$. The authors should explain why they consider such a correlation (with correlation coefficients around 0.6) to be satisfactory. It would be useful if the authors could show that the reduced correlation is not due to the simplifications mentioned in my point 1. To this end, the authors could simply perform a similar correlation analysis with the spilling factors with Gaussian kernel and show that the correlation coefficient does not improve significantly.

We consider the Spearman correlation coefficient of ~0.6 to be sufficient because it matches the value obtained using the ensemble method, which is a commonly used approach for estimating uncertainty in MLIPs. A similar approach was used by Goméz-Bombarelli et al. (10.1038/s41524-023-01180-8), who report comparable correlation values for the same database using trained potentials.

We believe we have addressed the KDE limitations with respect to spilling factor in the first comment and thus we expect that the simplification in the KDE method with regards to the spilling score is not the main cause of the reduced correlation. The reduced correlation might arise from the high similarity of chemical environments in the database. The testing structures are well represented in the training set, leaving little variation in similarity and dissimilarity between training and test points.

5) Fig. 4, left panel what is ϱ (without index) here? – average of ρk, minimal value of ρ_k, or simply ρ_k(i.e., one point in graph represents one atom)?

We thank the reviewer for pointing out the unclear aspect in Figure 4. We have added an index *k* to the ϱ in the left panel of Figure 4 and added more information to the caption.

6) In table 1 the authors show how their KDE density changes over a small series of similar systems described by a ML models of different quality. In this case, the KDE density values vary between 0.25 (bad description) to 0.85 (good description). Is it possible to define a threshold for a reasonable description? I know that this is not easy to say but a simple rule of thumb like 'any value below 0.5 is a clear indication of poor quality of simulation' _would be useful for practical applications.

Indeed, it is not easy to devise a robust threshold, but our experience does show that 0.5 typically indicates extrapolation in the ML model, which could serve as a rule of thumb. We have added this sentence to the MS to explicitly mention: "Furthermore, the KDE score values are on a similar scale to those reported in the case studies described above. Scores below 0.5 typically indicate an extrapolation, an observation that appears to be consistent across different types of ML models and showing that the KDE score of 0.5 could serve as a practical rule of thumb."

7) SI, Figure S1, S4, S5 and S9 – I do not think it is "Performance" what these plots really show (maybe correlation between ϱ and error in predicted forces?). Also, meaning of "SC" _(Spearman coefficient?) is not explained.

We thank the reviewer for the suggestion. We have updated and fixed the captions accordingly.

8) Figure S2, left panel - “maximum” _and “minimum” _curves are not shown.

Unfortunately, all three curves ("average", "maximum" and "minimum") are nearly identical (y ~ 1), and the curves overlap. We have modified the caption to make this clear.

Reviewer: 1

1) I wouldn’t call ρ an uncertainty, as uncertainties tend to put bounds around the output value (such as energy or forces). ρ is certainly a metric useful for identifying out-of-distribution atomic environments. In my opinion, ρ should be identified as such, rather than as a metric for uncertainty. Implementing this change would require a good amount of editing of the text and a change of title, but would not affect any of the results or conclusions of the work.

We agree with the reviewer that the density is not a direct uncertainty metric. In response, we have made minor edits throughout the MS and SI to clarify this point. At the same time, in principle, KDE density could be calibrated to yield specific uncertainty estimates, which could be explored in future work. Therefore, we assume that it is acceptable to relate the KDE density as metric for uncertainty in the current manuscript, but have added the following clarifying sentence to MS: "While this method does not provide quantitative uncertainty values, it offers a simple, general tool for probing out-of-distribution samples, which could potentially be calibrated for specific application to yield quantitative uncertainty values." Also, based on the second point of Reviewer one, we have made changes to both MS and SI to rename the density to score, which is a more accurate representation of the method.

2) The supporting information provides information on formulating the kernel bandwidth using the interquartile range (IQR) instead of the standard deviation, along with a brief discussion on why the standard deviation is preferred in this work. It would be useful to include a sentence or two in the main text pointing to the discussion in the SI to justify the use of standard deviation over IQR.

We have added a following sentence justifying the use of standard deviation over IQR to the MS: "This bandwidth estimation outperforms other common methods, such as using the interquartile range (IQR) or Silverman’s rule of thumb, which tend to underestimate the bandwidth, resulting in significantly lower values (see Section S1 for details)."

3) The value of k-nearest neighbors and number of dimensions for PCA were explored for reducing the MACE-MP0 descriptors of the rMD17 dataset. When moving to new descriptors and/or datasets, would the value of k and number of dimensions need to be tuned? It would be helpful to show a similar examination, at least for number of dimensions for the reduction of Al-centered SOAP descriptors since the original number of dimensions is so much higher than that of the MACE-MP0 descriptor. Though a comparison of PCA-SOAP (16) and SOAP (5470) is given in Table S1, a plot of descriptor size vs average score (once more values were explored) would

better indicate the effect of reducing the number of dimensions. It would also be interesting to see if the effect is the same for all 3 zeolites.

We do expect, based on our tests, that the optimal number of neighbors and minimal dimension of the PCA-reduced descriptor will depend on the specific dataset, a specific application and its requirements. However, a common observation is that this dependence is rather weak (see, e.g., Figure S4 and S6, and a new Figure S11). We address the issue of optimal selection of *k* parameter below (response to the Reviewer 2 comment #4) and herein focus on optimal selection of degree of dimensionality reduction extending our initial test on rMD17 dataset and foundational models descriptors to SOAP descriptors and the zeolite dataset for NMR.

As requested by the reviewer, we have plotted below (and added as a Figure S11 to SI with accompanying discussion) how the reduction in dimension of Al-centered SOAP descriptors influence the KDE density predictions. The Figure demonstrates that reduction to 16 dimensions yields consistent and reliable results, whereas further reduction leads to noticeable inaccuracies. This is, somewhat surprisingly, similar to the observation made for rMD17 dataset and foundational model descriptors (Figure S6) and supports our claim above about a rather weak dependence of the optimal dimensionality on a particular application. But clearly, all users of the approach would be well advised to test the generality of these observations for their systems.

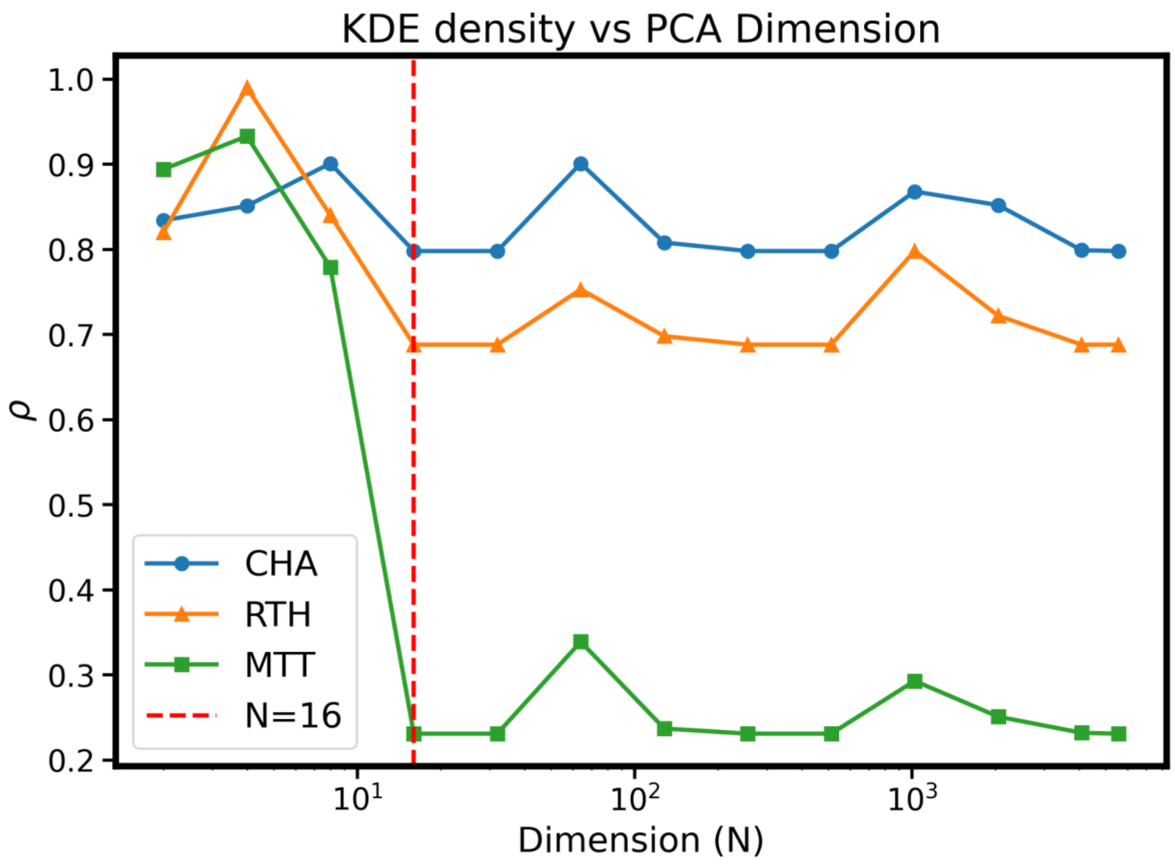

4) How is the optimal k determined? From Fig. S4, it appears that there are two dense regions of ρ near 0 and 1 at k=100. Is this the main criterion? If so, a histogram of ρ would be more useful to display than comparison against force errors.

Based on our tests, the choice of *k* does not significantly affect the KDE density calculation for the datasets considered. We selected k=100 because it provides a good coverage of the full range of ρ values from 0 to 1. For the rMD17 dataset, which we use as the benchmark throughout the paper, k=100 was found to be the most appropriate choice.

However, we admit the presentation in Figure S4 does not convey this message clearly and we thank the reviewer for a very good suggestion to plot rather the histogram of ρ. We have added the plot (below added as Figure S5 in SI), which presents histograms of ρ as a function of k points selected and significantly better supports our claim about k=100 being a suitable choice.

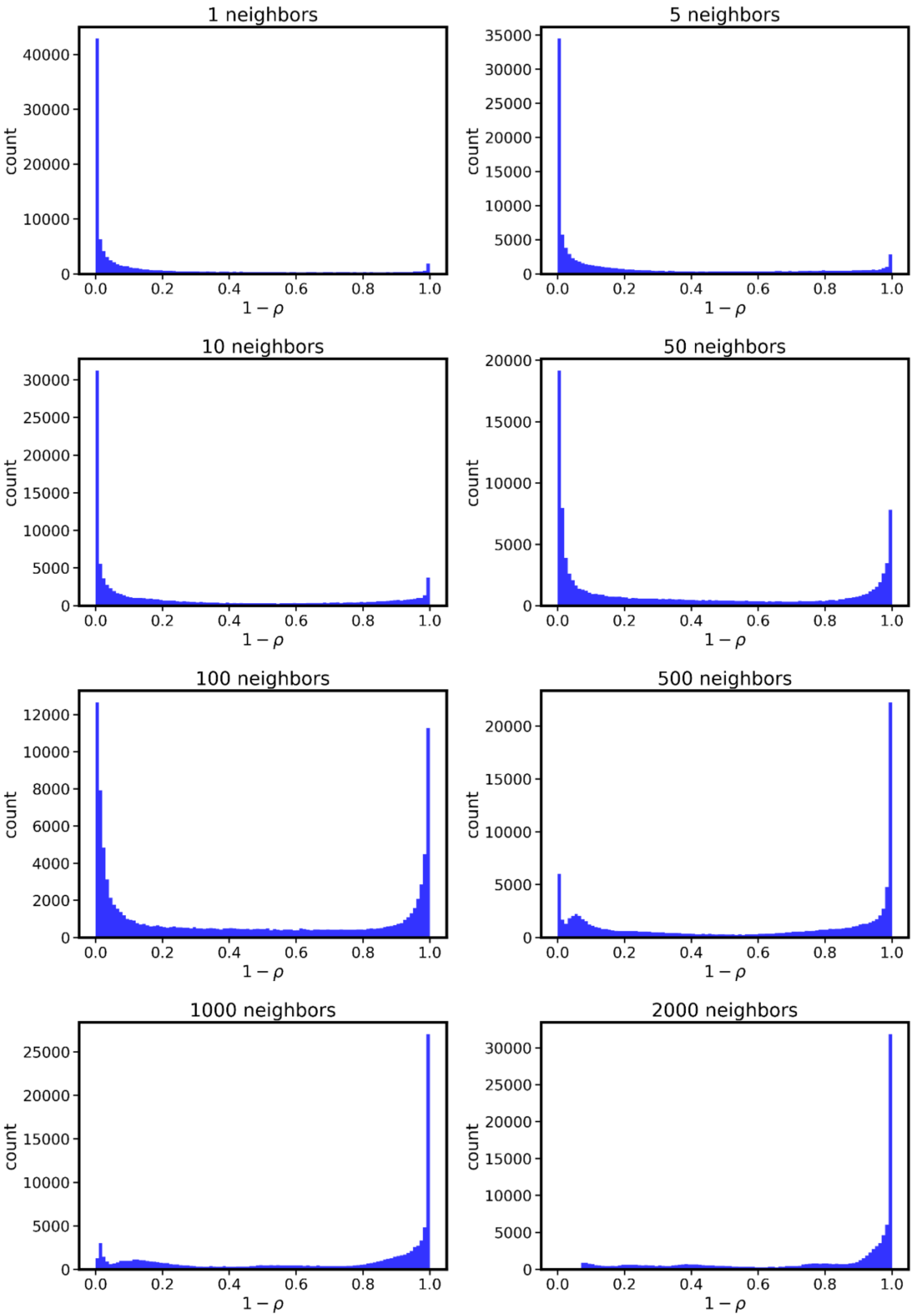

1 neighbors
5 neighbors
10 neighbors
50 neighbors
100 neighbors
500 neighbors
1000 neighbors
2000 neighbors
count
$1-\rho$

jz-2025-02595c.R2

Name: Peer Review Information for "A Simple and Scalable Kernel Density Approach for Reliable Uncertainty Quantification in Atomistic Machine Learning"

Second Round of Reviewer Comments

Reviewer: 1

Comments to the Author

The authors reasonably responded to my points.

Author's Response to Peer Review Comments:

Dear Editor,

we have addressed the non-scientific changes requested and uploaded the publication files without any markups.

Otherwise, we have not made any further changes to the manuscript.

With sincere regards,

Lukas Grajciar
